# The Impact of Permethrin and Cypermethrin on Plants, Soil Enzyme Activity, and Microbial Communities

**DOI:** 10.3390/ijms24032892

**Published:** 2023-02-02

**Authors:** Agata Borowik, Jadwiga Wyszkowska, Magdalena Zaborowska, Jan Kucharski

**Affiliations:** Department of Soil Science and Microbiology, Faculty of Agriculture and Forestry, University of Warmia and Mazury in Olsztyn, 10-719 Olsztyn, Poland

**Keywords:** insecticides, pyrethroids, *Zea mays*, rhizosphere diversity, microorganisms, soil enzymes

## Abstract

Pyrethroids are insecticides most commonly used for insect control to boost agricultural production. The aim of the present research was to determine the effect of permethrin and cypermethrin on cultured and non-cultivated bacteria and fungi and on the activity of soil enzymes, as well as to determine the usefulness of *Zea mays* in mitigating the adverse effects of the tested pyrethroids on the soil microbiome. The analyses were carried out in the samples of both soil not sown with any plant and soil sown with *Zea mays*. Permethrin and cypermethrin were found to stimulate the multiplication of cultured organotrophic bacteria (on average by 38.3%) and actinomycetes (on average by 80.2%), and to inhibit fungi growth (on average by 31.7%) and the enzymatic activity of the soil, reducing the soil biochemical fertility index (BA) by 27.7%. They also modified the number of operational taxonomic units (OTUs) of the *Actinobacteria* and *Proteobacteria* phyla and the *Ascomycota* and *Basidiomycota* phyla. The pressure of permethrin and cypermethrin was tolerated well by the bacteria *Sphingomonas* (clone 3214512, 1052559, 237613, 1048605) and *Bacillus* (clone New.ReferenceOTU111, 593219, 578257), and by the fungi *Penicillium* (SH1533734.08FU, SH1692798.08FU) and *Trichocladium* (SH1615601.08FU). Both insecticides disturbed the growth and yielding of *Zea mays*, as a result of which its yield and leaf greenness index decreased. The cultivation of *Zea mays* had a positive effect on both soil enzymes and soil microorganisms and mitigated the anomalies caused by the tested insecticides in the microbiome and activity of soil enzymes. Permethrin decreased the yield of its aerial parts by 37.9% and its roots by 33.9%, whereas respective decreases caused by cypermethrin reached 16.8% and 4.3%.

## 1. Introduction

Intensive development of the global economy has led to increased production and use of pesticides [1]. They serve as an important tool in crop protection and yield boosting [2] as approx. 45% of annual food production is lost due to invasions of agrophages [3], which are observed to develop increasing resistance to pesticides [1]. According to FAOSTAT data [4], insecticides account for 17.7% of the global use of pesticides.

One of the most commonly used classes of insecticides is represented by pyrethroids [5,6]. They are deployed to eradicate insects to increase production in agriculture [7,8] and forestry [9,10]. In addition, they are used in veterinary medicine, municipal landscape architecture, and households against ticks, mosquitoes, cockroaches, termites, fleas, and other pests [8]. Their use has increased significantly in the last decade due to the ban imposed on the application of phospho-organic insecticides, revealing higher acute toxicity [11]. Pyrethroids account for approximately 38% of the global insecticide market [12,13]. Permethrin is used to treat 9 to 10 million acres annually (out of 32–39 million acres treated with a mosquito adulticide) [14]. Although they are extremely important in pest control, their large-scale use contributes to the phenomenon of biomagnification [5,15], affecting human health and the natural environment [5,6,16]. Hence, their use should be permanently monitored to prevent their toxic effects [13,16,17].

Even though pyrethroids feature relatively low acute toxicity to humans and mammals [18,19], Andersen et al. [13] have emphasized that the Human Biomonitoring European Initiative (HBM4EU) has claimed them to be one of the 18 groups of substances raising serous concerns due to the growing exposure levels and insufficient knowledge on their potential adverse health effects. Pyrethroids enter the human body in various ways, including through the digestive system, respiratory system, and contact with the skin, manifested by paresthesia and gastrointestinal irritation [19,20,21,22]. The main routes by which pesticides contaminate the environment are drift, volatilization, leaching, and runoff [23].

These facts prompt the need to observe the response of the soil microbiome and soil enzymes to the pressure of these compounds. Admittedly, according to [24], microorganisms have the ability to biodegrade pesticides, which usually proceeds in three stages: (I) hydrolysis, oxidation, or reduction of the primary compound; (II) conjugation of phase I metabolites with sugar or amino acids to increase their solubility in water and produce less toxic metabolites; and (III) transformation of phase II metabolites to secondary conjugates [25,26]. In this case, hydrolysis of ester bonds is the fundamental process, whereas carboxylesterase plays a key role in pyrethroid degradation. Aminopeptidases and monooxygenases are also important in the cleavage of carboxylic ester bonds [18]. However, the response of microorganisms to insecticides may vary. On the one hand, these compounds promote the proliferation of, e.g., *Bacillus*, *Proteus*, *Corynebacterium*, *Streptomyces*, *Fusarium*, *Trichoderma*, or *Rhizopus*, responsible for the mineralization and availability of carbon, nitrogen, and phosphorus to plants [27], but on the other hand, cypermethrin significantly inhibits the abundance of certain other microorganisms (e.g., *Pseudomonas*, *Serratia*, *Staphylococcus*, *Nocardia*, *Micromonospora*, and *Aspergillus*), which ensure nutrient stabilization in the soil [27,28]. According to Shahid et al. [23], the responses of microorganisms correspond with growth inhibition, damages, as well as deformations of cells and their respiratory activity. Given that the microbiome–plant root interactions are based on a chemical dialogue, presumably, the sensitivity of microorganisms is strongly reflected in the responses of plants, including maize, to insecticides. Although *Zea mays* production is curbed by the environmental and biotic pressure exerted by pathogens and insects during cultivation and storage [29,30,31,32,33], it is not without reason that it is considered the most important crop worldwide, occupying an area of 160 million hectares of arable land, including over 90 million hectares cultivated in developing countries [30,32,34]. It is also worth emphasizing that despite its high adaptive abilities, good agricultural practices related to pesticide management urge for keeping high storage standards and not exceeding the maximum limits of insecticide residues in maize grain [35,36].

Insecticides are toxic by nature and are distributed in the environment, whereas their production, distribution, and use require regular biomonitoring [37,38,39]. This monitoring should extend not only to agricultural products [37,40,41], but also to the natural environment, including soils [18,42,43], water [37,44,45,46], and air [47]. A reliable indicator of the quality of soil ecosystems can be the measurement of the response of microbial communities and the activity of enzymes [48]. In addition, changes in the composition of the soil microbial communities and the activity of enzymes may be the first indicators of the adverse effects of pyrethroids in the soil [49,50,51]. The above reasons have prompted research into which soil microorganisms and enzymes would serve as indicators of soil quality under the pressure of pyrethroids. The aim of the present study was, therefore, to determine the effect of permethrin and cypermethrin on the number of cultured organotrophic bacteria, actinomycetes, and fungi. The impact of the applied pesticides on changes in the biodiversity of bacteria and fungi was also determined using the NGS method. The scale of potential inhibition of the activity of soil enzymes involved in the transformation of carbon, nitrogen, phosphorus, and sulfur was determined, including dehydrogenases, catalase, urease, acid phosphatase, alkaline phosphatase, arylsulfatase, and *β*-glucosidase. The effect of *Zea mays* on mitigating outcomes of the disorders caused by the tested pyrethroids in the soil microbiome was determined as well.

## 2. Results

### 2.1. Effect of Cypermethrin and Permethrin on Zea mays

The tested insecticides turned out to be toxic to *Zea mays* (Table 1). Permethrin decreased the yield of its aerial parts by 37.9% and its roots by 33.9%, whereas respective decreases caused by cypermethrin reached 16.8% and 4.3%. The results presented in Table 2, describing the greenness index of plants, still indirectly suggest the possibility of photosynthesis impairment by these insecticides.

### 2.2. Effect of Cypermethrin and Permethrin on the Activity of Soil Enzymes

**Figure 1:**
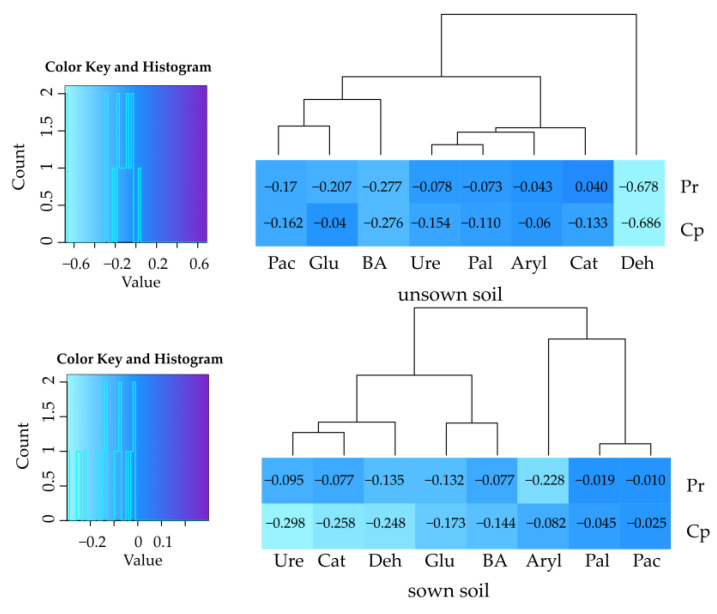
Index of the influence of insecticides (IF_in_) on soil enzyme activity. Deh—dehydrogenases; Cat—catalase; Ure—urease; Pac—acid phosphatase; Pal—alkaline phosphatase; Aryl—arylsulfatase; Glu—*β*-glucosidase; Pr—soil contaminated with permethrin; Cp—soil contaminated with cypermethrin; BA—index of soil biochemical fertility.

The activity of soil enzymes was found to depend on soil utilization, insecticide type, and enzyme type (Table S1). Only the activity of dehydrogenases (from 13.5% to 68.6%) was inhibited in both soil types by permethrin and cypermethrin. Both insecticides significantly impaired the activity of phosphatases in the unsown soil, while such an effect was not observed in the sown soil. In the sown soil type, both permethrin and cypermethrin inhibited activities of catalase by 7.7% and 25.8%, urease by 9.5% and 29.8%, arylsulfatase by 22.8% and 8.2%, and *β*-glucosidase by 13.2% and 17.3%, respectively, whereas in the bare soil, activities of catalase (by 13.3%) and urease (by 15.4%) were suppressed only by cypermethrin. Both insecticides significantly reduced the value of the biochemical index of soil fertility (BA). The index of insecticide effect (IF_in_) on the activity of soil enzymes attained negative values, indicating an inhibitory effect of these preparations (Figure 1).

**Figure 2:**
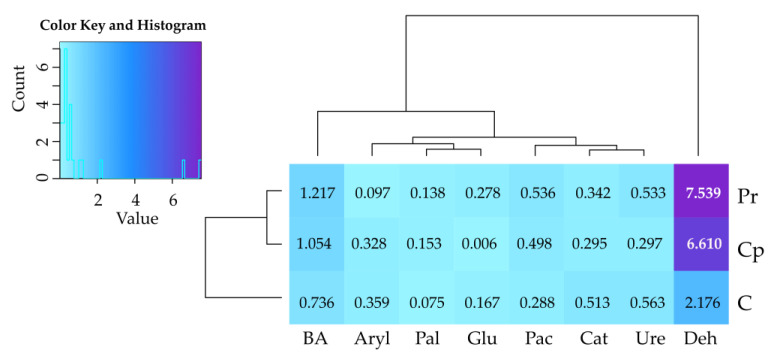
Index of the influence of *Zea mays* (IF_Zm_) on the activity of soil enzymes. Deh—dehydrogenases; Cat—catalase; Ure—urease; Pac—acid phosphatase; Pal—alkaline phosphatase; Aryl—arylsulfatase; Glu—*β*-glucosidase; C—soil uncontaminated; Pr—soil contaminated with permethrin; Cp—soil contaminated with cypermethrin; BA—index of soil biochemical fertility.

Soil cropping with *Zea mays* enhanced activities of all enzymes except for arylsulfatase in the soil contaminated with permethrin and *β*-glucosidase in the soil contaminated with cypermethrin (Figure 2). In the soil contaminated with permethrin, *Zea mays* stimulated the activity of dehydrogenases by 753.9%, acid phosphatase by 53.6%, urease by 53.3%, catalase by 34.2%, *β*-glucosidase by 27.8%, and alkaline phosphatase by 13.8%; in the soil contaminated with cypermethrin, *Zea mays* increased the activity dehydrogenases by 661.0%, acid phosphatase by 49.8%, arylsulfatase by 32.8%, urease by 29.7%, catalase by 29.5%, and alkaline phosphatase by 15.3%.

### 2.3. Effect of Cypermethrin and Permethrin on Soil Microorganisms

#### 2.3.1. Cultured Microorganisms

**Figure 3:**
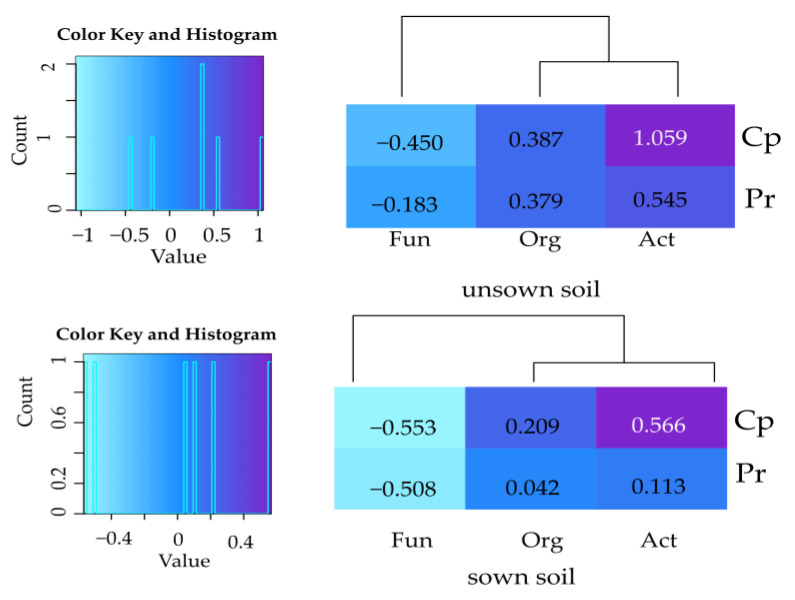
Index of the influence of insecticides (IF_in_) on the number of microorganisms. Org—organotrophic bacteria; Act—actinomycetes; Fun—fungi; Pr—soil contaminated with permethrin; Cp—soil contaminated with cypermethrin.

**Figure 4:**
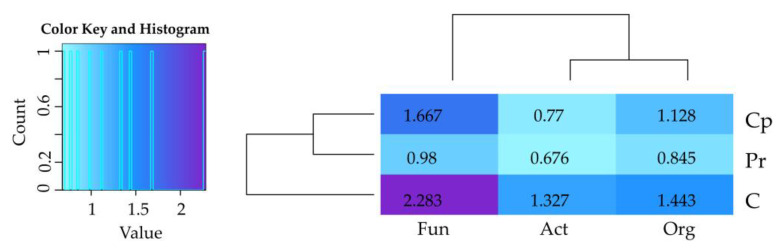
Index of the influence of *Zea mays* (IF_Zm_) on the number of microorganisms. Org—organotrophic bacteria; Act—actinomycetes; Fun—fungi; C—soil uncontaminated; Pr—soil contaminated with permethrin; Cp—soil contaminated with cypermethrin.

The values of the indices of insecticide effect (IF_in_) on the proliferation of microorganisms in objects contaminated with permethrin (Pr) and cypermethrin (Cp) (Figure 3) were significantly lower compared to the IF_Zm_ values (Figure 4). 

The index of IF_in_ attained positive values in the case of organotrophic bacteria and actinobacteria as well as negative ones in the case of fungi. Changes in the abundance of microorganisms in the soil were affected to a greater extent by cypermethrin than by permethrin. A stronger effect of both insecticides on organotrophic bacteria and actinobacteria was observed in the bare soil than in the soil sown with *Zea mays* (Table S2). An opposite effect was noted in the case of fungi, i.e., higher negative values of IF_in_ were noted in the sown soil. In the soil used as black fallow, a significant shift in the proliferation rate occurred only in the case of actinobacteria under the effect of cypermethrin, as indicated by the CD value (Table S3). This insecticide caused a significant decrease in CD values of all microorganisms in the soil sown with *Zea mays*. In the soil cropped with this cereal, the CD values decreased as well but only for organotrophic bacteria and fungi. The effect of *Zea mays* on the values of the CD index differed from the effects induced by the insecticides tested. Its cultivation increased the CD value computed for organotrophic bacteria by 32%, for actinobacteria by 60%, and for fungi by 25%. This was, however, not reflected in the ecophysiological diversity (EP) of these microorganisms as the EP value in the soil sown with *Zea mays* increased only for organotrophic bacteria, decreased only for actinobacteria, and did not change for fungi (Table S4). In this experimental series, the ecophysiological diversity of organotrophic bacteria and actinobacteria was diminished only by permethrin.

The positive values of the IF_Zm_ index (Figure 4) proved that *Zea mays* cultivation contributed significantly to increased abundance of organotrophic bacteria, actinobacteria, and fungi in the soil. The positive impact of the crop on cultured microorganisms was the strongest in the control soil not contaminated with insecticides, significantly weaker in the soil with cypermethrin, and the weakest in the soil contaminated with permethrin.

#### 2.3.2. Bacteria Determined with the NGS Method

**Figure 5:**
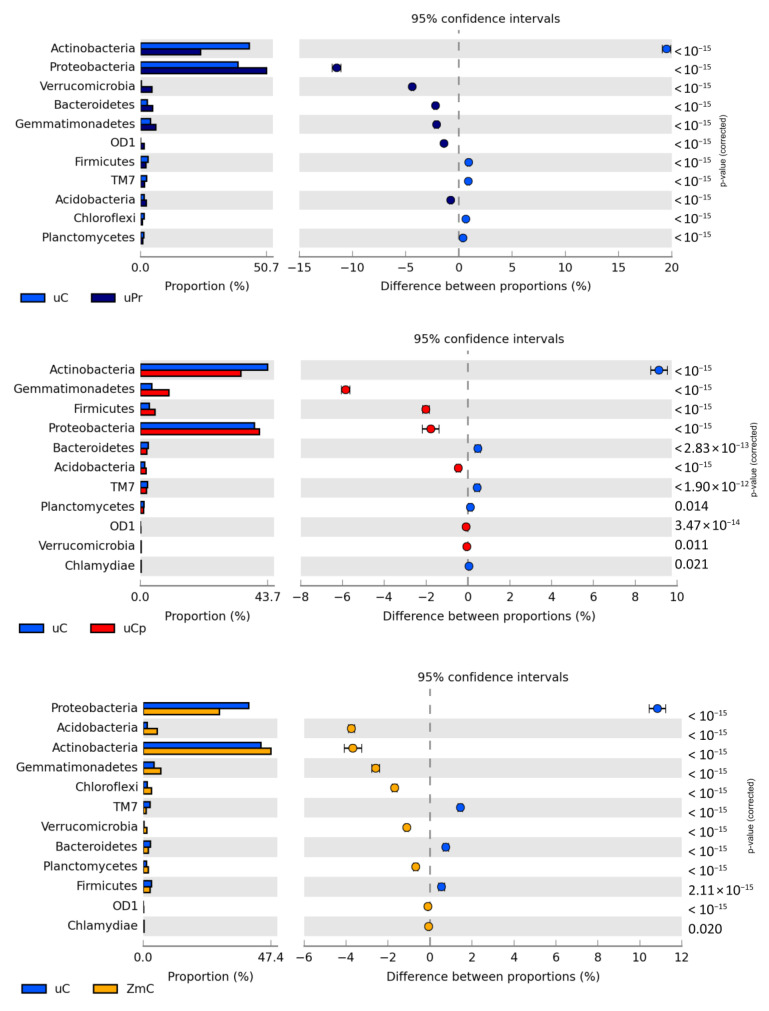

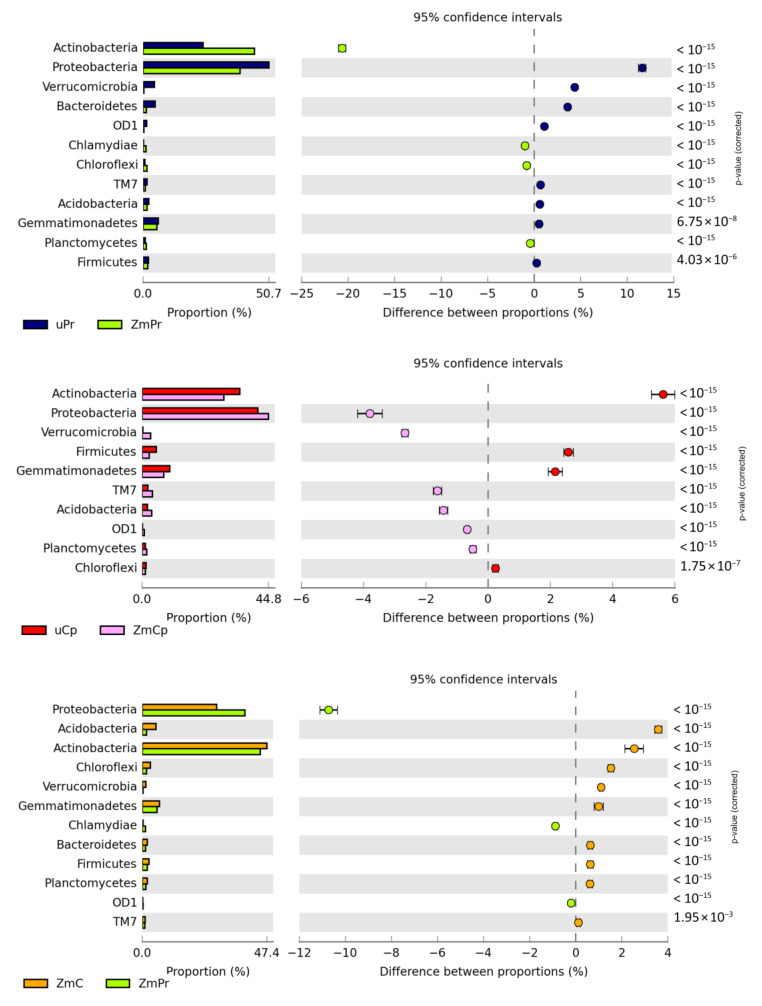

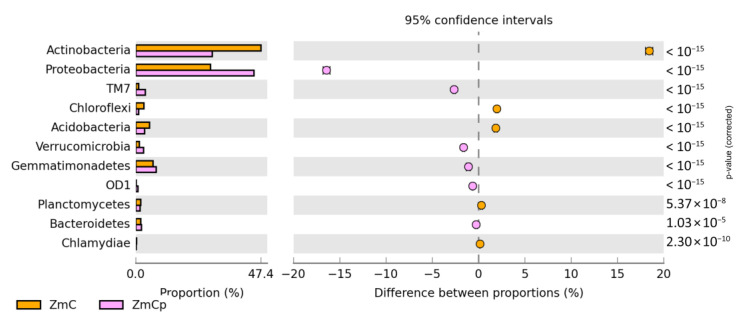
Relative abundance of dominant phylum bacteria in the soil. uC—contaminated unsown soil; uPr—sown soil contaminated with permethrin; uCp—sown soil contaminated with cypermethrin; ZmC—uncontaminated soil sown with *Zea mays*; ZmPr—soil sown with *Zea mays* contaminated with permethrin; ZmCp—soil sown with *Zea mays* contaminated with cypermethrin.

Both the unsown soil and the soil sown with *Zea mays* were mainly colonized by bacteria from the phylum *Actinobacteria*, followed by those from the phylum *Proteobacteria* (Figure 5). The cultivation of *Zea mays* contributed to a 3.67% increase in the OTU number of the first mentioned phylum and a 10.85% decrease in the OTU number of the latter phylum. Contamination of the bare soil with cypermethrin decreased the OTU number of *Actinobacteria* by 9.14% and increased that of *Gemmatimonadetes* by 5.85%, whereas its contamination with permethrin reduced the OTU number of the first phylum by as much as 19.5% and increased the OTU number of *Proteobacteria* by 11.49%. Additionally, in the soil sown with *Zea mays*, cypermethrin was observed to reduce the OTU number of the phylum *Actinobacteria* (by 18.43%) and increase that of the phylum *Proteobacteria* (by 16.42%). In turn, permethrin only increased the OTU number of the phylum *Proteobacteria* (by 10.72%).

**Figure 6:**
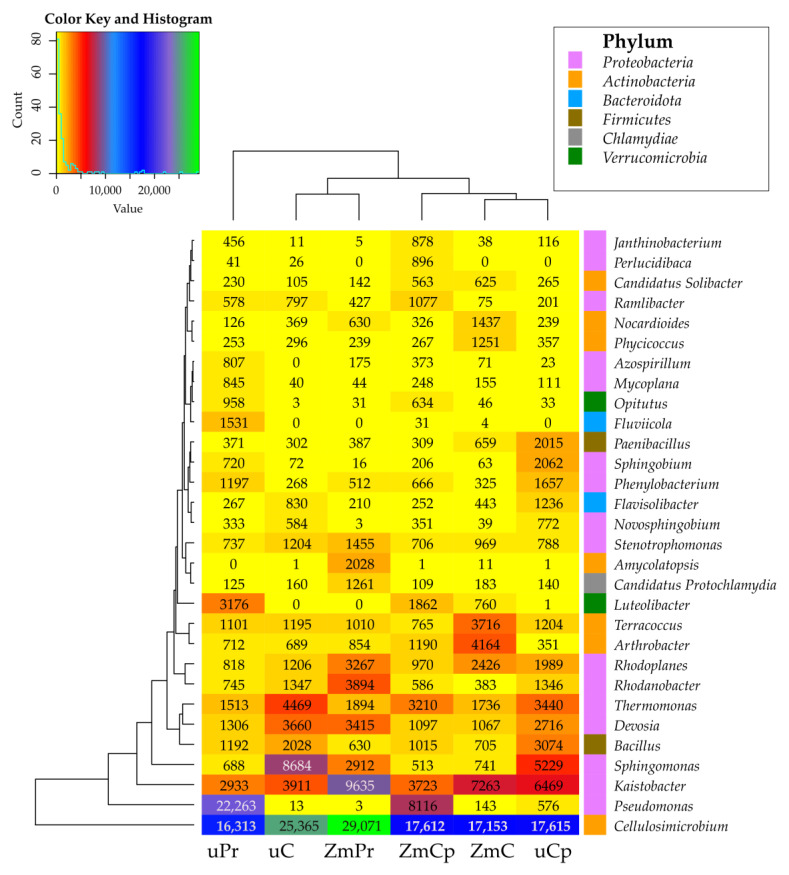
The relative abundance of the dominant bacterial genus in the soil. uC—contaminated unsown soil; uPr—sown soil contaminated with permethrin; uCp—sown soil contaminated with cypermethrin; ZmC—uncontaminated soil sown with *Zea mays*; ZmPr—soil sown with *Zea mays* contaminated with permethrin; ZmCp—soil sown with *Zea mays* contaminated with cypermethrin.

The genera prevailing in the analyzed soil samples regardless of soil utilization and contamination with insecticides, determined based on the average OTU number, turned out to be *Cellulosimicrobium*, *Kaistobacter*, *Pseudomonas*, *Sphingomonas*, *Thermomonas*, *Devosia*, *Rhodoplanes*, *Terracoccus*, *Bacillus*, *Rhodanobacter*, and *Arthrobacter*. However, in the bare soil not contaminated with insecticides, the highest OTU numbers were determined for *Cellulosimicrobium* and *Sphingomonas*; in the soil contaminated with permethrin, for *Pseudomonas* and *Cellulosimicrobium*; and in the soil contaminated with cypermethrin, for *Cellulosimicrobium*, *Kaistobacter*, and *Sphingomonas*. In the soil cropped with *Zea mays* and not contaminated with insecticides, the prevailing phyla turned out to be *Cellulosimicrobium* and *Arthrobacter*. In the sown soil, permethrin and cypermethrin decreased the OTU number of *Arthrobacter*, whereas permethrin increased the OTU number of *Cellulosimicrobium*. In the unsown soil, both insecticides decreased the OTU number of bacteria from the genus *Cellulosimicrobium* (Figure 6).

**Figure 7:**
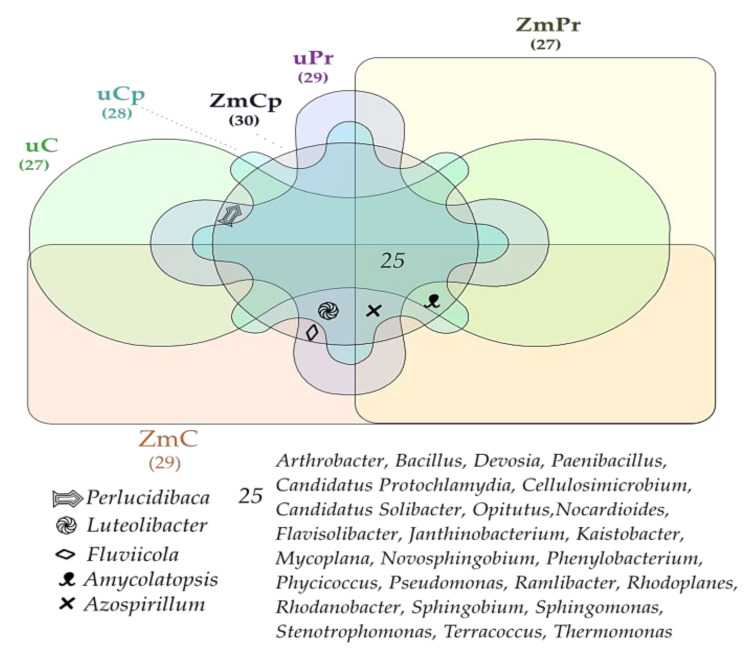
Venn diagram showing common and unique types of bacteria in the soil. uC—contaminated unsown soil; uPr—sown soil contaminated with permethrin; uCp—sown soil contaminated with cypermethrin; ZmC—uncontaminated soil sown with *Zea mays*; ZmPr—soil sown with *Zea mays* contaminated with permethrin; ZmCp—soil sown with *Zea mays* contaminated with cypermethrin.

Despite changes in OTU abundance of the mentioned genera, the Venn diagram proved relative stability of bacterial communities in the soil, regardless of its utilization mode and contamination with permethrin and cypermethrin, as indicated by the very rich core bacteriome consisting of as many as 25 genera of the 30 identified ones (Figure 7).

**Figure 8:**
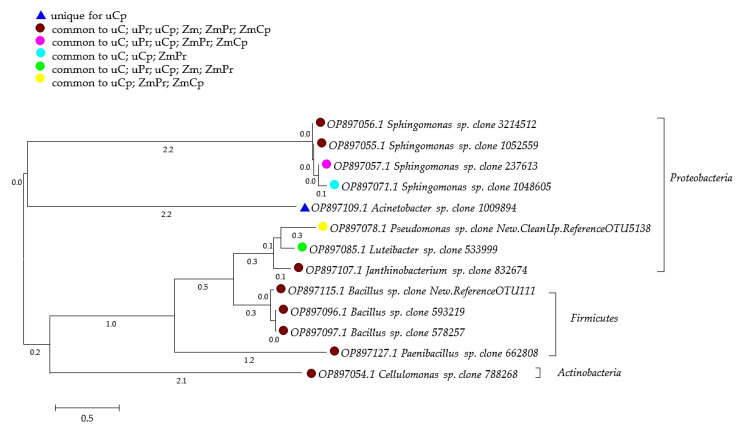
Phylogenetic tree of bacteria isolated from the soil made using the neighbor-joining method. uC—contaminated unsown soil; uPr—sown soil contaminated with permethrin; uCp—sown soil contaminated with cypermethrin; ZmC—uncontaminated soil sown with *Zea mays*; ZmPr—soil sown with *Zea mays* contaminated with permethrin; ZmCp—soil sown with *Zea mays* contaminated with cypermethrin.

All soils samples were abundantly populated by *Arthrobacter*, *Bacillus*, *Candidatus*, *Protochlamydia*, *Candidatus*, *Solibacter*, *Cellulosimicrobium*, *Devosia*, *Flavisolibacter*, *Janthinobacterium*, *Kaistobacter*, *Mycoplana*, *Nocardioides*, *Novosphingobium*, *Opitutus*, *Paenibacillus*, *Phenylobacterium*, *Phycicoccus*, *Pseudomonas*, *Ramlibacter*, *Rhodanobacter*, *Rhodoplanes*, *Sphingobium*, *Sphingomonas*, *Stenotrophomonas*, *Terracoccus*, and *Thermomonas*. The dominant bacteria identified in the analyzed soil samples were *Sphingomonas* (clone 3214512, 1052559, 237613, 1048605) and *Bacillus* (clone New.ReferenceOTU111, 593219, 578257) (Figure 8). The highest OTU number (8482) of the first mentioned bacteria clone was found in the soil not contaminated with insecticides and not sown with *Zea mays*. The contamination of bare soil with cypermethrin reduced its OTU number to 4830, and with permethrin, to 444. In turn, *Zea mays* cultivation contributed to its OTU number increase to 2150 in the soil treated with permethrin and to its OTU number decrease to 361 in the soil amended with cypermethrin. The remaining clones of *Bacillus* predominated in the bare soil contaminated with insecticides.

#### 2.3.3. Fungi Determined with the NGS Method

**Figure 9:**
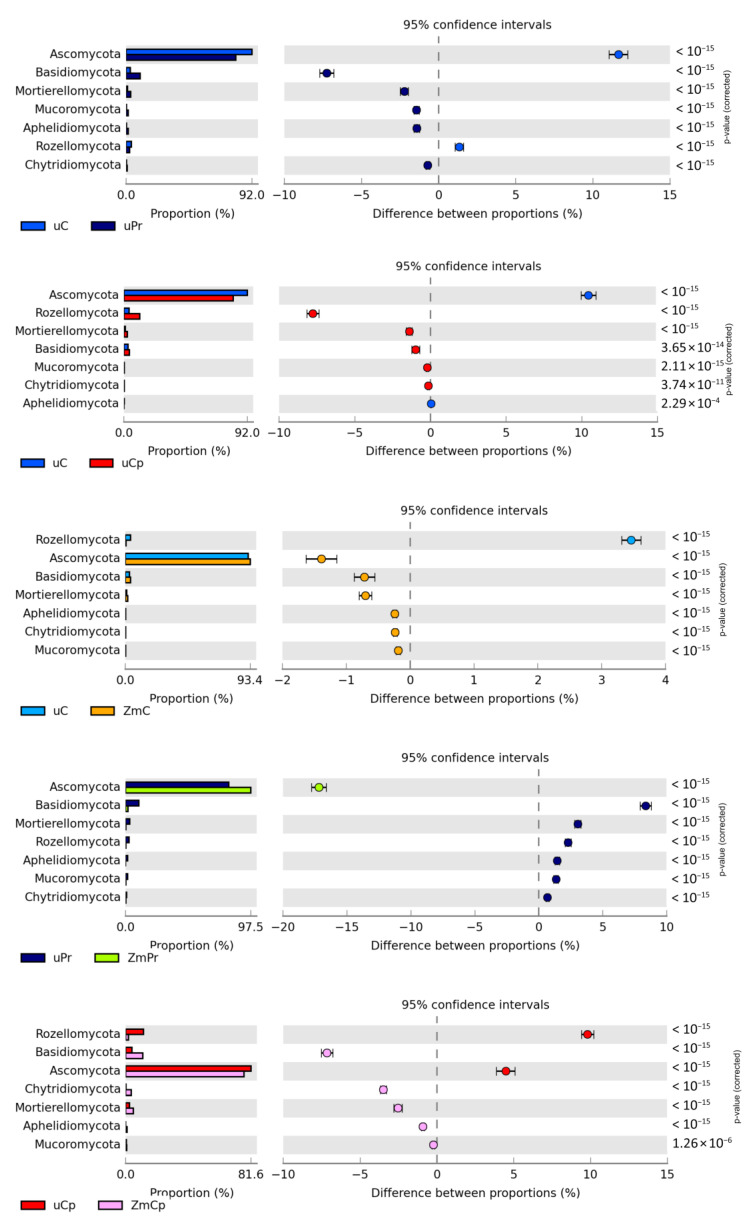

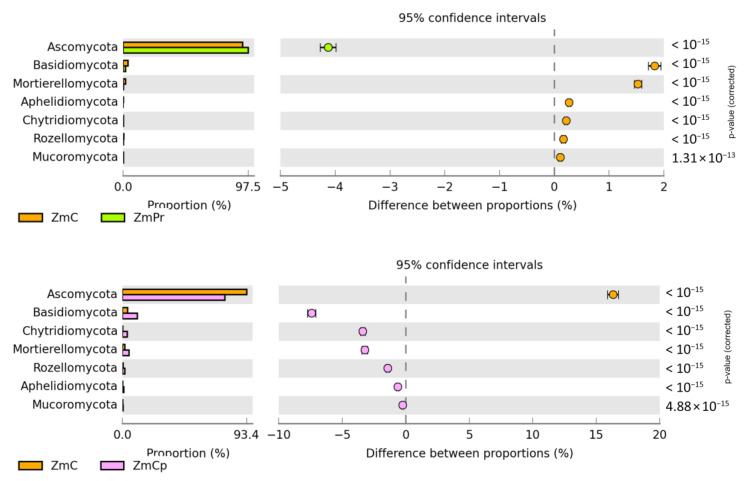
Relative abundance of dominant phylum fungi in the soil. uC—contaminated unsown soil; uPr—sown soil contaminated with permethrin; uCp—sown soil contaminated with cypermethrin; ZmC—uncontaminated soil sown with *Zea mays*; ZmPr—soil sown with *Zea mays* contaminated with permethrin; ZmCp—soil sown with *Zea mays* contaminated with cypermethrin.

The prevailing fungi identified in the tested soil were those from the phylum *Ascomycota*. In the case of this phylum, 92.02% OTUs were identified in the bare soil and 93.42% in the soil sown with *Zea mays*. Crop cultivation caused a 1.39% increase in the OTU number of *Ascomycota* and a 3.47% decrease in the OTU number of *Rozellomycota*. Differences observed in the case of the other phyla were small and did not exceed 1%. The contamination of bare soil with permethrin decreased the OTUs of *Ascomycota* by 11.67% and increased the OTUs of *Basidiomycota* by 7.25%. Cypermethrin also had a negative impact on the structure of *Ascomycota*, decreasing their OTU number by 10.45%. Soil treatment with this insecticide did not cause such changes in *Basidiomycota* as its treatment with permethrin did, while it increased the OTU number of the phylum *Rozellomycota* by 7.77%. Likewise, in the bare soil and in the soil cropped with *Zea mays*, only cypermethrin affected the structure of the phylum *Ascomycota*, by reducing the number of identified OTUs by 16.32%, whereas its effect on the other phyla was contrary to that observed in the bare soil, i.e., it increased OTU numbers of *Basidiomycota* (by 7.41%), *Chytridiomycota* (by 3.38%), and *Mortierellomycota* (by 3.23%). The effect of permethrin on fungi was weaker compared to that of cypermethrin as it increased the OTU number of the phylum *Ascomycota* by 4.13% and reduced OTUs of *Basidiomycota* by 1.83% and *Monoblepharomycota* by 1.53% (Figure 9).

**Figure 10:**
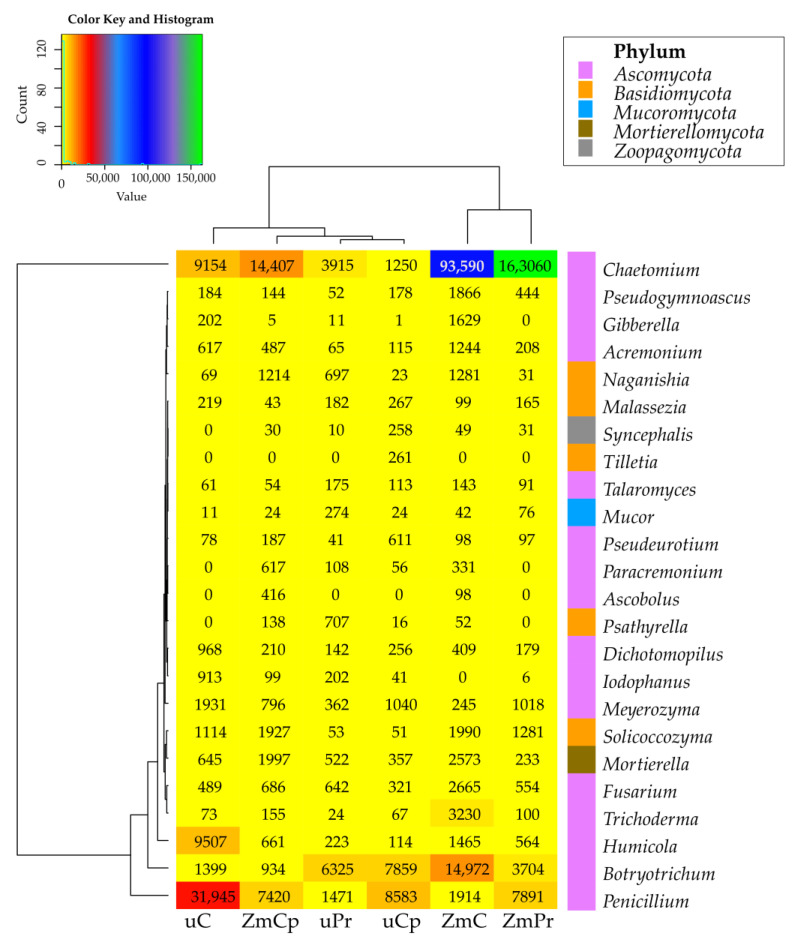
The relative abundance of the dominant type of fungus in the soil. uC—contaminated unsown soil; uPr—sown soil contaminated with permethrin; uCp—sown soil contaminated with cypermethrin; ZmC—uncontaminated soil sown with *Zea mays*; ZmPr—soil sown with *Zea mays* contaminated with permethrin; ZmCp—soil sown with *Zea mays* contaminated with cypermethrin.

The prevailing genera of fungi in the soil tested, regardless of its utilization and treatment with pyrethroids, turned out to be representatives of the phylum *Ascomycota*: *Chaetomium* (average number of OTU 47,563), *Penicillium* (9871 OTU), *Botryotrichum* (5866 OTU), and *Humicola* (2089 OTU); phylum *Basidiomycota*: *Solicoccozyma* (1069); and phylum *Mortierellomycota*: *Mortierella* (1055 OTU). The sowing of uncontaminated soil with *Zea mays* increased the OTU number of the identified fungal genera by 118% on average. The effects of pyrethroids differed depending on the soil utilization mode. In the bare soil, permethrin and cypermethrin decreased the mean OTU number by 73% and 63%, respectively, while in the cropped soil, permethrin increased OTU abundance by 38%, and cypermethrin decreased it by 75%. The cultivation of *Zea mays* positively affected the proliferation of fungi from the following genera: *Chaetomium*, *Botryotrichum*, *Trichoderma*, *Fusarium*, *Mortierella*, *Acremonium*, *Pseudogymnoascus*, *Gibberella*, and *Naganishia*. In contrast, it adversely affected the proliferation of fungi representing *Penicillium*, *Meyerozyma*, *Humicola*, *Dichotomopilus*, *Malassezia*, and *Meyerozyma*. Regardless of soil utilization mode, permethrin and cypermethrin decreased OTU numbers of most isolated fungal genera. Only in the bare soil was permethrin observed to increase OTU numbers of *Botryotrichum*, *Mucor*, *Naganishia*, *Psathyrella*, and *Talaromyces*, and cypermethrin to increase OTU numbers of *Botryotrichum*, *Syncephalis*, and *Tilletia*. In turn, in the soil cropped with *Zea mays*, permethrin increased the OTU numbers of *Chaetomium*, *Penicillium*, *Meyerozyma*, and *Malassezia*, whereas cypermethrin increased the OTU numbers of *Penicillium*, *Meyerozyma*, and *Ascobolus* (Figure 10).

**Figure 11:**
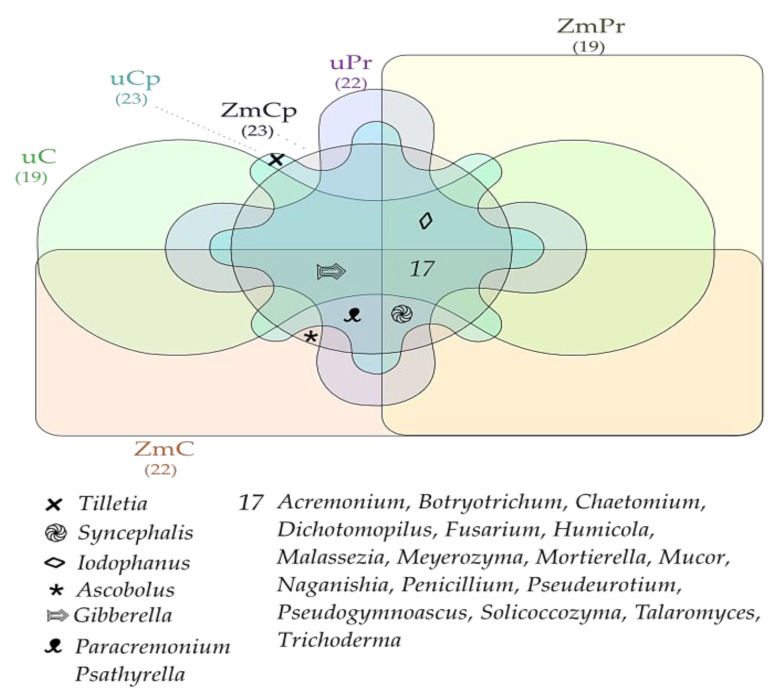
Venn diagram showing common and unique types of fungi in the soil. uC—contaminated unsown soil; uPr—sown soil contaminated with permethrin; uCp—sown soil contaminated with cypermethrin; ZmC—uncontaminated soil sown with *Zea mays*; ZmPr—soil sown with *Zea mays* contaminated with permethrin; ZmCp—soil sown with *Zea mays* contaminated with cypermethrin.

**Figure 12:**
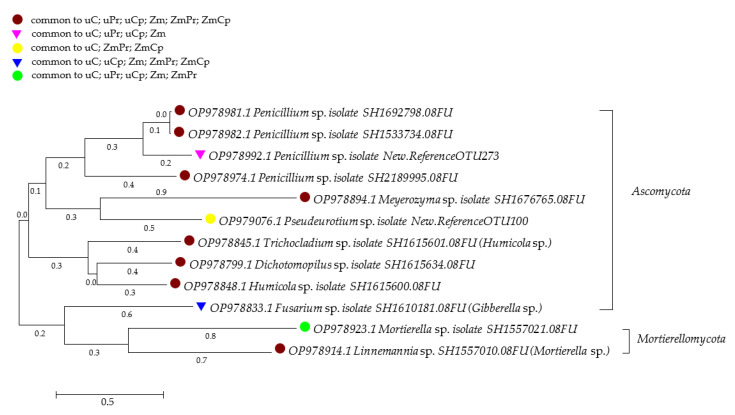
Phylogenetic tree of fungi isolated from the soil by the neighbor-joining method. uC—contaminated unsown soil; uPr—sown soil contaminated with permethrin; uCp—sown soil contaminated with cypermethrin; ZmC—uncontaminated soil sown with *Zea mays*; ZmPr—soil sown with *Zea mays* contaminated with permethrin; ZmCp—soil sown with *Zea mays* contaminated with cypermethrin.

The Venn diagram showed that, regardless of permethrin and cypermethrin application, the core mycobiome of both bare soil and soil cropped with *Zea mays* included fungi from the genera: *Acremonium*, *Botryotrichum*, *Chaetomium*, *Dichotomopilus*, *Fusarium*, *Humicola*, *Malassezia*, *Meyerozyma*, *Mortierella*, *Mucor*, *Naganishia*, *Penicillium*, *Pseudeurotium*, *Pseudogymnoascus*, *Solicoccozyma*, *Talaromyces*, and *Trichoderma* (Figure 11). The dominant fungi identified in the bare unsown soil not contaminated with insecticides turned out to be representatives of isolates of *Penicillium* (isolate SH1692798.08FU, SH2189995.08FU, New Reference OTU273) and *Humicola* (SH1615600.08FU), but in the bare unsown soil exposed to the pressure of permethrin and cypermethrin, the best conditions for development were offered to *Penicillium* (SH1533734.08FU), and in the cropped soil, to *Penicillium* (SH1692798.08FU, SH1533734.08FU) and *Trichocladium* (SH1615601.08FU) (Figure 12).

## 3. Discussion

### 3.1. Effect of Pesticides on Plants

Although pyrethroids are claimed to be more environment-friendly than other insecticides [7,8,52], their mass-scale use to aid food production leads to their accumulation in the soil, which ultimately affects the growth and development of plants [53,54,55]. In the present study, soil contamination with permethrin and cypermethrin impaired the growth and development of *Zea mays* by inducing oxidative stress in plants, which generates radical oxygen species and triggers the breakdown of proteins and chlorophyll pigments [18,24,56]. These unbeneficial phenomena diminish photosynthesis yield [57]. Bashir et al. [52] and Duran et al. [55] proved that long-term exposure to pyrethroid insecticides contributed to a high rate of lipid peroxidation, inducive to degradation of the cell membrane systems, which promotes ROS generation and impairs the basic biochemical and physiological processes in plants.

### 3.2. Effect of Pesticides on the Activity of Soil Enzymes

The effect of pyrethroids may be assessed by analyzing changes in the activity of enzymes affecting nutrient metabolism and, by the same means, also soil health and quality [49,58,59]. In the present study, both permethrin and cypermethrin decreased the value of the biochemical index of soil fertility (BA), being an indicator of activities of enzymes from the class of oxidoreductases and hydrolases [60]. Furthermore, Filimon et al. [61] and Tejada et al. [49] demonstrated the negative impact of cypermethrin on the recorded values of activities of dehydrogenases, urease, catalase, and phosphatase. In turn, Zhuang et al. [62] reported that beta-cypermethrin applied in doses of 10–80 mg kg^−^^1^ of soil had no significant effect on the activity of dehydrogenases, ureases, acid phosphatase, and *β*-glucosidase. The impact of pyrethroids on the biological activity of soil is strongly determined by the organic matter content because soil enzymes penetrate into humic-enzymatic complexes, modifying their resistance to thermal denaturation and proteolytic degradation [59,63,64]. Differences in enzymatic activity may also stem from a broad spectrum of factors determining degradation rate and mobility of pyrethroids and their metabolites in the soil, including their adsorption and desorption [65].

### 3.3. Effect of Pesticides on Soil Microorganisms

Excessive and uncontrolled use of insecticides triggers changes in the biodiversity of the entire ecological system [25,66]. Tejada et al. [49] as well as Shahid and Khan [58] have emphasized that the effect of pesticides, and insecticides in particular, on cultured soil microorganisms and activity of soil enzymes has not yet been thoroughly identified. Hence, investigations that enable the observation of changes in populations of important autochthonous microorganisms are highly valuable [67], the more thatthe impact of insecticides on soil microbiota is not one-dimensional. In the present study, permethrin and cypermethrin stimulated the proliferation of organotrophic bacteria and actinobacteria, and inhibited that of fungi. In turn, in the study conducted by Das et al. [68], the use of permethrin and cypermethrin reduced the population numbers of ammonifying and nitrifying bacteria, and enhanced the proliferation of non-symbiotic bacteria fixing N_2_. This is significantly related to the fact that pyrethroids undergo various processes in the soil, e.g., transformation, sorption, or oxidation [69].

The stimulating effect of permethrin and cypermethrin on organotrophic bacteria and actinobacteria, demonstrated in the present study, is strictly correlated with the capability of autochthonous microorganisms for using pyrethroids as a carbon source and their degradation to such inorganic compounds as carbon dioxide and water [66]. Inorganic compounds formed upon biomineralization are absorbed by microorganisms and plants as nutrients or play the role of electron acceptors in the respiratory chain [47]. A stronger effect of cypermethrin on changes in the population numbers of cultured microorganisms compared to permethrin is probably due to the presence of an additional chiral center possessing a cyano group, being typical of type II pyrethroids such as cypermethrin [70].

Complex assessment of pesticide effect on soil health should take account of the changes taking place in communities of soil microorganisms [26,53]. This is especially important given the fact that insecticides may accumulate in the top 15 cm soil layer [49], where microorganisms exhibit the highest activity [71]. In the present study, both the bare soil and the soil cropped with *Zea mays* were mainly colonized by bacteria from the phylum *Actinobacteria*, followed by those from the phylum *Proteobacteria*. Permethrin and cypermethrin modified the OTU numbers of bacteria from the *Actinobacteria* and *Proteobacteria* phyla as well as OTU numbers of fungi from the *Ascomycota* and *Basidiomycota* phyla. Special attention was paid to the bacteria from the *Cellulosimicrobium*, *Kaistobacter*, *Pseudomonas*, *Sphingomonas*, *Thermomonas*, *Devosia*, *Rhodoplanes*, *Terracoccus*, *Bacillus*, *Rhodanobacter*, and *Arthrobacter* genera, as well as to fungi from the *Chaetomium*, *Penicillium*, *Botryotrichum*, *Humicola*, *Solicoccozyma*, and *Mortierella* genera, which were the most abundant in the soil samples tested. Tejada et al. [49] suggested that the impact of pyrethroids on soil quality might be evaluated by analyzing changes in communities of soil microbiota. After soil treatment with cypermethrin, Zhang et al. [72] observed a modification in its microbiota composition. They noted that the most abundant bacteria representing the phylum *Firmicutes* were replaced by *Bacteroidetes* bacteria or γ-*proteobacteria*. Shahid and Khan [58] have emphasized that microorganisms abundant in the rhizosphere play an important role in pesticide degradation and stress mitigation, which ultimately lead to the restoration of soil quality. It is among the autochthonous microorganisms exposed to the pressure of pyrethroids that bacteria and fungi strains suitable for biomineralization should be searched for [47]. According to data from the literature [15,47,69,73,74], numerous bacteria and fungi are capable of pyrethroid biomineralization. The most potent in this respect include *Achromobacter*, *Acidomonas*, *Acinetobacter*, *Azoarcus*, *Bacillus*, *Brevibacterium*, *Catellibacterium*, *Clostridium*, *Lysinibacillus*, *Micrococcus*, *Ochrobactrum*, *Pseudomonas*, *Rhodococcus*, *Serratia*, *Sphingobium*, *Sphingomonas*, *Sphingopyxis*, and *Streptomyces* bacteria and strains of fungi from the *Acremonium*, *Aspergillus*, *Candida*, *Cladosporium*, *Microsphaeropsis*, *Penicillium*, *Trichoderma*, and *Westerdykella* genera [69]. Such a broad spectrum of microorganisms involved in the degradation of pyrethroid insecticides is due to the fact that permethrin serves as a source of carbon and cypermethrin as a source of carbon and nitrogen to multiple microorganisms [75].

In the present study, unlike soil amendment with pyrethroids, cropping with *Zea mays* had a positive effect on the soil microbiome. In addition, it alleviated the adverse effects of permethrin and cypermethrin on bacteria and fungi. This effect of *Zea mays* may be ascribed to its anatomical and physiological features, including especially the production and secretion of organic compounds into soil [76]. These properties allow recommending *Zea mays* for the phytoremediation of soils under pesticide pressure, as earlier emphasized by Erguven and Koçak [77].

## 4. Materials and Methods

### 4.1. Soil

The research was carried out on soil taken from arable land in the northern part of the Olsztyn Lake District (NE Poland, 53.72° N, 20.42° E). According to the grain size classification of the International Union of Soil Sciences and the United States Department of Agriculture [78], the soil was loamy sand containing 1.69% of the fraction <0.002 mm (clay), 20.13% of the fraction 0.002–0.05 mm (silt), and 78.18% of the fraction 0.05 mm –2.0 mm. The physicochemical and chemical properties of the soil were as follows: pH_KCl_—6.3; hydrolytic acidity (HAC)—1.16 cmol(+)g kg^−1^; sum of exchangeable base cations (EBC)—5.82 cmol(+)g kg^−1^; cation exchange capacity (CEC)—6.98 cmol(+)g kg^−1^; alkaline cation saturation (ACS)—83.4%; C_org_ content—7.5 g kg^−1^; N_tot_—0.64 g kg^−1^; available P—98 mg kg^−1^; available K—133 mg kg^−1^; and available Mg—42 mg kg^−1^. All soil properties were presented per d.m. of soil. A detailed description of methods and equipment used for physicochemical and chemical analyses of soil was provided in our previous work [79].

### 4.2. Insecticides

Two pyrethroid insecticides were used in the study: permethrin (Pr) and cypermethrin (Cp). Permethrin is a type I pyrethroid [80], whereas cypermethrin is a type II pyrethroid with an α-cyano group [70,81]. Both are second-generation insecticides [69]. Permethrin was applied to the soil in the form of an Aspermet 200 EC preparation, whereas cypermethrin was applied in the form of an Arpon G preparation.

Aspermet 200 EC (Asplant-Skotniccy Sp. J, Jaworzno, Poland) is used to control ticks, flies, pharaoh ants, cockroaches, bed bugs, and silverfish. An amount of 1 dm^3^ of this preparation contains 200 g of permethrin (C_21_H_20_Cl_2_O_3_), 3-Phenoxybenzyl 3-(2,2-dichlorovinyl)-2,2-dimethylcyclopropanecarboxylate. The half-life (t½) of permethrin in soil ranges from 5 to 55 days [69]_._

Arpon G (Laboratorios Zotal S.L., Camas, Seville, Spain) is used against ticks, mosquitoes, and cockroaches. An amount of 1 dm^3^ of this preparation contains 100 g cypermethrin (C_22_H_19_Cl_2_NO_3_), [Cyano-(3-phenoxyphenyl)methyl]3-(2,2-dichloroethenyl)-2,2-dimethylcyclopropane-1-carboxylate. The half-life (t½) of cypermethrin in soil ranges from 14.4 to 105.8 days [69].

### 4.3. Study Design

This study was conducted in a greenhouse in the following research variants: (1) bare uncontaminated soil (uC); (2) bare soil contaminated with permethrin (uPr); (3) bare soil contaminated with cypermethrin (uCp); (4) uncontaminated soil cropped with *Zea mays* (ZmC); (5) soil cropped with *Zea mays* and contaminated with permethrin (ZmPr); and (6) soil cropped with *Zea mays* contaminated with cypermethrin (ZmCp). Each research variant was prepared in 4 replications. The experiment was established in polyethylene pots, each filled with 3 kg of loamy sand sieved through a screen with a mesh size of 5 mm. Before the pots had been filled with soil, it was mixed with NPKMg fertilizers and in variants No. 2 and 5, additionally with permethrin, and in variants No. 3 and 6, with cypermethrin. Permethrin and cypermethrin were applied in doses of 0 and 80 mg per 1 kg^−1^ d.m. of soil. Their dose was established based on data from the literature [10,24,69] and considering the possibility of contaminating areas of municipal architecture during the common and repeated use of these pyrethroids for tick control. The soil was fertilized with 150 mg N kg^−1^ soil in the form of CO(NH_2_)_2_, 50 mg P in the form of KH_2_PO_4_, and 150 mg K in the form of KH_2_PO_4_ + KCl, as well as with 20 mg Mg kg^−1^ soil in the form of MgSO_4_ × 7H_2_O. In variants No. 4, 5, and 6, soil was sown with 8 seeds of *Zea mays* of LG 32.58 cultivar (variety registered in the European Union), and 4 plants were left in each pot after the emergence stage. The soil in variants No. 1, 2, and 3 remained bare, whereas that from variants No. 4, 5, and 6 was sown with *Zea mays* to analyze its phytoremediation potential, whose biomass may afterward be intended for energy production based on green chemistry methods [82]. *Zea mays* is one of the key crops grown worldwide [82,83,84,85,86]. Throughout the experimental period (60 days), soil moisture content was kept stable at 60% m.w.c. using distilled water. Daylength ranged from 15 h 13 min to 16 h 35 min. The mean air temperature ranged from 17.9 °C to 19.8 °C, whereas the mean relative humidity reached 77%. At the 4th leaf development stage (BBCH—Biologische Bundesanstalt, Bundessortenamt and Chemical Scale 14) and at the 9th leaf stage (BBCH 19), the leaf greenness index SPAD (Soil and Plant Analysis Development) was determined using a Chlorophyll Meter 2900P *SPAD 502* (KONICA MINOLTA, Inc., Chiyoda, Japan). At the BBCH 51 stage, the plants were cut, the root and above ground green mass (both fresh weight and dry weight) was determined, and soil samples were collected for further laboratory analyses.

### 4.4. Soil Microbiological Analysis

#### 4.4.1. Culture Microbes

Microorganisms were isolated with the serial dilution method following the procedure described in our earlier work [87]. The fresh soil samples, carefully mixed and sieved through a screen with 2 mm mesh diameter, were determined for the number of organotrophic bacteria on the Bunt and Roviry’s medium [88], actinobacteria on the Kuster and Williams’s medium with nystatin and actidione addition [89], and fungi on the Martin’s medium [90]. Determinations were conducted in 4 replications for each experimental variant. The cultures of microorganisms were grown in an incubator (PSelecta Incudigit, Barcelona, Spain) at a temperature of 28 °C. The colony forming units (cfu) of microorganisms were presented as per 1 kg d.m. of soil.

#### 4.4.2. Isolation of DNA and Identification of Bacteria and Fungi with the NGS Method

DNA was isolated from the soil samples using “Genomic Mini AX Bacteria+” (A&A Biotechnology), with universal primers 1055F (5′-ATGGCTGTCGTCAGCT-3′) and 1392R (5′-ACGGGCGGTGTGTAC-3′) amplifying the fragment of a bacterial 16S rRNA gene and ITS. PCR conditions were provided in detail in our previous works [45,46]. Once libraries had been prepared, the sequencing of the genetic material based on the hypervariable region V3–V4 of the 16S rRNA gene and the ITS1 fragment was performed in an Illumina MiSeq sequencer Illumina MiSeq (Genomed S.A., Warsaw, Poland). The selected region was amplified, and a library was developed with the use of specific primers 341F (5′-CCTACGGGNGGCWGCAG-3′) and 785R (5′-GACTACHVGGGTATCTAATCC-3′) (bacteria), as well as sequences of ITS1FI2 (5′-GAACCWGCGGARGGATCA-3′) and 5.8S (5′-CGCTGCGTTCTTCATCG-3′) (fungi). Demultiplexed read-outs were analyzed with the cutadapt v. 2.10 software (Dortmund, Germany). The sequences were aligned using a fast-join algorithm, unclust, whereas sequence chimeras were removed using the usearch61 algorithm. Bioinformatic analysis was conducted using the QIIME (Quantitative Insights Into Microbial Ecology) package based on reference sequence database Greengenes v13_8, an Illumina modification, and that of fungi, based on the UNITE database. The manuscript presents data for relative abundance (phylum, genus) with OTU > 1%. Sequences of bacteria and fungi were deposited in GenBank NCBI under access numbers Prokaryotic 16S rRNA https://www.ncbi.nlm.nih.gov/nuccore/?term=OP914644:OP916021[accn] accessed on 4 December 2022 and https://www.ncbi.nlm.nih.gov/nuccore/?term=OP897054:OP897145[accn] accessed on 2 December 2022, Eukaryotic Nuclear rRNA/ITS: https://www.ncbi.nlm.nih.gov/nuccore/?term=OP978693:OP979103[accn] accessed on 14 December 2022.

### 4.5. Biochemical Analyses of Soil

Dehydrogenases (EC 1.1.), catalase (EC 1.11.1.6), urease (EC 3.5.1.5), alkaline phosphatase (EC 3.1.3.1), acid phosphatase (EC 3.1.3.2), arylsulfatase (EC 3.1. 6.1), and *β*-glucosidase (EC 3.2.1.21) were analyzed using standard methods presented in the study by Borowik et al. [91] and Zaborowska et al. [92]. Immediately after soil sample collection from pots, activity of the above mentioned enzymes was determined in 4 replications. The enzymatic activity was presented per 1 kg d.m. of soil and 1 h, and expressed in the following units: dehydrogenases—µmol TFF (triphenyl formazan); catalase—mol O_2_; urease—mmol N-NH_4_; alkaline phosphatase, acidic phosphatase, arylsulfatase, and *β*-glucosidase—mmol PNP (p-nitrophenol). A detailed description (substrates, products, buffers, temperatures, incubation times, reaction arrestment time, and wavelength used to determine activities of soil enzymes) was provided in our previous work [93]. Activities of all enzymes, except for catalase, were determined using a Perkin-Elmer spectrophotometer Lambda 25 (Waltham, MA, USA), catalase activity was measured using a potassium permanganate titration method.

### 4.6. Data and Statistical Analysis

The numbers of the abovementioned groups of cultured microorganisms were used to compute the colony development index (CD) and the index of ecophysiological diversity of microorganisms (EP) [94]. To compute CD and EP values, grown colonies of microorganisms were counted every day for 10 consecutive days. The results of activity determination of seven soil enzymes (Deh, Cat, Ure, Pal, Pac, Aryl, and Glu) served to calculate the value of the biochemical indicator of soil quality (BA) according to the formula provided in a work by Wyszkowska et al. [60]. These data were developed statistically using Statistica 13.3 [95]. The normality of data distribution was verified with the Shapiro–Wilk test and the Kruskal–Wallis test, and then the results were compared with the post-hoc Tukey test. Homogeneous groups were counted separately for sown and unsown soil. Formulas developed by Wyszkowska et al. [50] were also used to compute the indices of the effect of permethrin, cypermethrin, and *Zea mays* on the count of cultured microorganisms and activity of soil enzymes.

All graphical data were presented after eliminating OTUs lower than 1% compared to the total OTU number. Bacterial and fungal phyla were compared statistically using the G-test (w/Yates’) + Fisher test, by means of the STAMP 2.1.3 software [96]. Bacterial and fungal genera with taxonomic affiliation to the phylum were presented in the form of a heat map prepared using the RStudio v1.2.5033 software [97] with a gplots library [98] and R core environment [99]. Data of unique and common bacterial and fungal genera were visualized using the InteractiVenn software for cluster analysis [100].

The phylogenetic tree of bacteria and fungi was prepared using the neighbor-joining method. Evolutionary analyzes were conducted in MEGA7 [101,102].

## 5. Conclusions

Permethrin and cypermethrin applied to soil in a dose of 80 mg kg^−1^ of soil were observed to inhibit the activity of soil enzymes, stimulate the proliferation of cultured bacteria, and inhibit the proliferation of fungi. In addition, they decreased the OTU number of non-cultured predominating bacteria from the phylum *Actinobacteria* and fungi from the *Ascomycota* and *Basidiomycota* phyla as well as increasing the abundance of bacteria from the phylum *Proteobacteria*. Despite these disproportions, the study failed to identify bacteria and fungi unique to the analyzed independent variables; however, its results prove that active strains suitable for bioaugmentation should be searched for among the bacteria *Sphingomonas* and *Bacillus* and among the fungi *Penicillium* and *Trichocladium*. Maize cultivation improved the condition of the soil microbiome and increased the colony development index (CD) of organotrophic bacteria, actinobacteria, and fungi. It contributed to an increase in the number of OTUs of *Actinobacteria* and a decrease in the number of OTUs of *Proteobacteria*. *Zea mays* cultivation increased the number of *Ascomycota* OTUs and the soil biochemical fertility index (BA). Moreover, even though soil contamination with permethrin and cypermethrin adversely affected its growth and development, the effects achieved in mitigating the unbeneficial outcomes of these pyrethroid insecticides on microbiological and biochemical soil properties substantiate considering *Zea mays* as an effective phytoremediation crop. The conducted research also became the matrix for the next experiment, in which both the soil and plants of the *Poaceae* family will be exposed to changing weather conditions in the field.

## Figures and Tables

**Table 1:** Yield of aerial parts and roots of *Zea mays*, g d.m. pot^−1^.

Object	Aerial Parts	Roots
ZmC	53.411 ^a^	6.176 ^a^
ZmPr	33.173 ^c^	4.082 ^c^
ZmCp	44.411 ^b^	5.913 ^b^

Homogeneous groups according to yield of *Zea mays* (for aerial parts and roots separately) denoted with identical letters (^a–c^). ZmC—uncontaminated soil sown with *Zea mays*; ZmPr—soil sown with *Zea mays* contaminated with permethrin; ZmCp—soil sown with *Zea mays* contaminated with cypermethrin.

**Table 2:** *Zea mays* leaf greenness index (SPAD).

Object	Development Phase	
	Four Leaves	Six Leaves
ZmC	45.313 ^b^	39.681 ^a^
ZmPr	44.081 ^c^	37.644 ^ab^
ZmCp	46.981 ^a^	36.628 ^b^

Homogeneous groups according to *Zea mays* leaf greenness index (for fourth and sixth leaves separately) denoted with identical letters (^a–c^). ZmC—uncontaminated soil sown with *Zea mays*; ZmPr—soil sown with *Zea mays* contaminated with permethrin; ZmCp—soil sown with *Zea mays* contaminated with cypermethrin.

## Data Availability

Data are available by contacting the authors.

## Supplementary Materials

The supporting information can be downloaded at: https://www.mdpi.com/article/10.3390/ijms24032892/s1.

## Abbreviations

The following abbreviations are used in this manuscript:

uC—contaminated unsown soil; uPr—sown soil contaminated with permethrin; uCp—sown soil contaminated with cypermethrin, ZmC—uncontaminated soil sown with *Zea mays*; ZmPr—soil sown with *Zea mays* contaminated with permethrin; ZmCp—soil sown with *Zea mays* contaminated with cypermethrin; SPAD—the leaf greenness index; Org—organotrophic bacteria; Act—actinomycetes; Fun—fungi; Deh—dehydrogenases; Cat—catalase; Ure—urease; Pac—acid phosphatase; Pal—alkaline phosphatase; Aryl—arylsulfatase, Glu—*β*-glucosidase; CD—colony development index; EP—ecophysiological diversity index; BA—the soil biochemical fertility index.
