# Supplementary material for: The Impact of Permethrin and Cypermethrin on Plants, Soil Enzyme Activity, and Microbial Communities
Source: Int J Mol Sci. 2023 Feb 2;24(3):2892. doi: 10.3390/ijms24032892 (PMC9917378; doi:10.3390/ijms24032892)
Supplement: Supplementary file 1 [file ijms-24-02892-s001.zip › ijms-2138952-supplementary.pdf]

International Journal of
Molecular Sciences

MDPI

Supplementary Materials

The Impact of Permethrin and Cypermethrin on Plants, Soil Enzyme Activity, and Microbial Communities

Agata Borowik, Jadwiga Wyszkowska *, Magdalena Zaborowska and Jan Kucharski

Department of Soil Science and Microbiology; University of Warmia and Mazury in Olsztyn; Faculty of Agriculture and Forestry; Poland; agata.borowik@uwm.edu.pl (A.B.); m.zaborowska@uwm.edu.pl (M.Z.); jan.kucharski@uwm.edu.pl (J.K.)

* Correspondence: jadwiga.wyszkowska@uwm.edu.pl

Table S1. Enzyme activity in 1 kg d.m. soil in 1 hour.

Object	Deh	Cat	Ure	Pac	Pal	Aryl	Glu	BA
				Unsown soil				
uC	1.388 [a]	0.115 [a]	0.173 [a]	3.026 [a]	0.586 [a]	0.085 [a]	0.294 [a]	5.666 [a]
uPr	0.446 [b]	0.119 [a]	0.159 [a]	2.513 [b]	0.543 [ab]	0.081 [a]	0.233 [b]	4.095 [b]
uCp	0.436 [b]	0.099 [b]	0.146 [a]	2.537 [b]	0.522 [b]	0.080 [a]	0.282 [a]	4.102 [b]
				Sown soil				
ZmC	4.408 [a]	0.174 [a]	0.270 [a]	3.898 [a]	0.630 [a]	0.115 [a]	0.343 [a]	9.838 [a]
ZmPr	3.812 [b]	0.160 [a]	0.244 [ab]	3.860 [a]	0.618 [ab]	0.089 [c]	0.298 [b]	9.081 [b]
ZmCp	3.315 [c]	0.129 [b]	0.189 [c]	3.801 [a]	0.601 [b]	0.106 [b]	0.284 [b]	8.425 [c]

Homogeneous groups denoted with letters [(a–c)] were counted for each enzyme and BA separately for sown soil and unsown soils, Deh - dehydrogenases, µmol TFF; Cat - catalase, mol O_2; Ure - urease, mmol N-NH_4; Pac - acid phosphatase, mmol PN; Pal - alkaline phosphatase, mmol PN; aryl - arylsulfatase, mmol PN; Glu - *β*-glucosidase, mmol PN; uC – contaminated unsown soil; uPr – sown soil contaminated with permethrin; uCp - sown soil contaminated with cypermethrin; ZmC – uncontaminated soil sown with *Zea mays*; ZmPr – soil sown with *Zea mays* contaminated with permethrin; ZmCp - soil sown with *Zea mays* contaminated with cypermethrin; BA - index of soil biochemical fertility.

Table S2. Number of microorganisms in 1 kg d.m. soil.

Object	Org, $\times 10^9$	Act, $\times 10^9$	Fun, $\times 10^6$
		Unsown soil	
uC	11.829 [b]	10.286 [c]	28.053 [a]
uPr	16.317 [a]	15.896 [b]	22.910 [a]
uCp	16.411 [a]	21.180 [a]	15.429 [b]
		Sown soil	
ZmC	28.894 [b]	23.938 [c]	92.106 [a]
ZmPr	30.110 [b]	26.650 [b]	45.352 [b]
ZmCp	34.925 [a]	37.497 [a]	41.144 [b]

Homogeneous groups denoted with letters [(a–c)] were counted for each group of microorganisms and separately for sown soil and unsown soils, Org—organotrophic bacteria; Act—actinomycetes; Fun—fungi; uC – contaminated unsown soil; uPr – sown soil contaminated with permethrin; uCp - sown soil contaminated with cypermethrin; ZmC – uncontaminated soil sown with *Zea mays*; ZmPr – soil sown with *Zea mays* contaminated with permethrin; ZmCp - soil sown with *Zea mays* contaminated with cypermethrin.

Table S3. Colony development index (CD) of microorganisms.

Object	Org	Act	Fun
Unsown soil			
uC	30.049 [ab]	16.824 [b]	38.230 [a]
uPr	28.490 [b]	16.009 [b]	44.084 [a]
uCp	31.964 [a]	27.532 [a]	41.717 [a]
Sown soil			
ZmC	39.746 [a]	26.853 [a]	47.813 [a]
ZmPr	31.294 [b]	27.348 [a]	31.452 [b]
ZmCp	30.966 [b]	23.994 [b]	35.624 [b]

Homogeneous groups denoted with letters [(a–c)] were counted for each group of microorganisms and separately for sown soil and unsown soils, Org—organotrophic bacteria; Act—actinomycetes; Fun—fungi; uC – contaminated unsown soil; uPr – sown soil contaminated with permethrin; uCp - sown soil contaminated with cypermethrin; ZmC – uncontaminated soil sown with *Zea mays*; ZmPr – soil sown with *Zea mays* contaminated with permethrin; ZmCp - soil sown with *Zea mays* contaminated with cypermethrin.

Table S4. Ecophysiological diversity index (EP) of microorganisms.

Object	Org	Act	Fun
Unsown soil			
uC	0.825 [c]	0.903 [a]	0.674 [b]
uPr	0.848 [b]	0.898 [a]	0.772 [a]
uCp	0.980 [a]	0.911 [a]	0.695 [b]
Sown soil			
ZmC	0.938 [a]	0.803 [c]	0.671 [a]
ZmPr	0.892 [b]	0.853 [b]	0.635 [a]
ZmCp	0.912[ab]	0.960 [a]	0.692 [a]

Homogeneous groups denoted with letters [(a–c)] were counted for each group of microorganisms and separately for sown soil and unsown soils, Org—organotrophic bacteria; Act—actinomycetes; Fun—fungi; uC – contaminated unsown soil; uPr – sown soil contaminated with permethrin; uCp - sown soil contaminated with cypermethrin; ZmC – uncontaminated soil sown with *Zea mays*; ZmPr – soil sown with *Zea mays* contaminated with permethrin; ZmCp - soil sown with *Zea mays* contaminated with cypermethrin.
